# Sex differences in the utilization and outcomes of endovascular treatment after acute ischemic stroke: A systematic review and meta-analysis

**DOI:** 10.3389/fgwh.2022.1032592

**Published:** 2023-01-18

**Authors:** Menglu Ouyang, Sultana Shajahan, Xiaoying Liu, Lingli Sun, Cheryl Carcel, Katie Harris, Craig S. Anderson, Mark Woodward, Xia Wang

**Affiliations:** ^1^ The George Institute for Global Health, Faculty of Medicine, University of New South Wales, Sydney, NSW, Australia; ^2^ Stroke Division, The George Institute for Global Health, Beijing, China; ^3^ Sydney School of Public Health, Sydney Medical School, The University of Sydney, Sydney, NSW, Australia; ^4^ Neurology Department, Royal Prince Alfred Hospital, Sydney Health Partners, Sydney, NSW, Australia; ^5^ The George Institute for Global Health, School of Public Health, Imperial College London, London, United Kingdom

**Keywords:** sex difference, endovascular treatment, ischemic stroke, female, treatment

## Abstract

**Background:**

Studies of sex differences in the use and outcomes of endovascular treatment (EVT) for acute ischemic stroke report inconsistent results

**Methods:**

We systematically searched PubMed and Embase databases for studies examining sex-specific utilization of EVT for acute ischemic stroke published before 31 December 2021. Estimates were compared by study type: randomized clinical trials (RCTs) and non-RCTs (hospital-based, registry-based or administrative data). Random effects odds ratios (ORs) were generated to quantify sex differences in EVT use. To estimate sex differences in functional outcome on the modified Rankin scale after EVT, the female:male ratio of ORs and 95% confidence intervals (CIs) were obtained from ordinal or binary analysis.

**Results:**

6,396 studies were identified through database searching, of which 594 qualified for a full review. A total of 51 studies (36 non-RCT and 15 RCTs) reporting on sex-specific utilization of EVT were included, and of those 10 estimated the sex differences of EVT on functional outcomes. EVT use was similar in women and men both in non-RCTs (OR: 1.03, 95% CI: 0.96–1.11) and RCTs (1.02, 95% CI: 0.89–1.16), with consistent results across years of publication and regions of study, except that in Europe EVT treatment was higher in women than men (1.15, 95% CI: 1.13–1.16). No sex differences were found in the functional outcome by either ordinal and binary analyses (ORs 0.95, 95% CI: 0.68–1.32] and 0.90, 95% CI: 0.65–1.25, respectively).

**Conclusions:**

No sex differences in EVT utilization or on functional outcomes were evident after acute ischemic stroke from large-vessel occlusion. Further research may be required to examine sex differences in long-term outcomes, social domains, and quality of life.

**Systematic Review Registration:**

https://www.crd.york.ac.uk/PROSPERO/display_record.php?RecordID=226100, identifier: CRD42021226100.

## Introduction

Stroke is the second leading cause of death and the third leading cause of disability, consequently causing considerable suffering and economic and social burden worldwide (1). Evidence suggests that there are sex differences in the association between major risk factors and the incidence of stroke. Hypertension, smoking and atrial fibrillation are more strongly associated with increased risk of stroke in women compared to men (2). Sex also impacts stroke outcomes. Women have worse functional recovery and quality of life after stroke compared to men due to poorer health at the time of stroke onset, advanced age, and greater severity compared to men (3, 4). From a large population-based study conducted in Australia, women were more likely to arrive at hospital by ambulance, but less likely to receive stroke care management prior to hospital admission than men (5). However, the data on the aspects of accessibility and quality of clinical treatment that may contribute to worse outcomes in women are still scarce (4), there is a need to understand potential sex differences in treatment outcomes (6).

Endovascular treatment (EVT) is a guideline-recommended therapy for acute ischemic stroke (AIS) due to large-vessel occlusion (LVO) (7–11). Compared to the standard medical management with intravenous thrombolysis, in which intravenous (IV) recombinant tissue plasminogen activator (rtPA) is used within the first 4.5 h of symptom onset, EVT can be applied in a long time window (≥6 h) (12), to increase the odds of disability-free survival, and improved quality of life, life expectancy, and cost of treatment (13–16). A recent meta-analysis of studies examining sex-specific rates for IV-rtPA found that women were 13% less likely to receive treatment than men (17). Despite the rising utilization of EVT for AIS, it is unclear whether sex differences exist in this patient group and of any impact on outcomes in real-world populations (6, 18, 19). Furthermore, an individual patient data meta-analysis of randomized controlled trials (RCT) from the Highly Effective Reperfusion Using Multiple Endovascular Devices (HERMES) collaboration indicates that sex does not modify the treatment effect of EVT, showing women and men benefit equally (14, 20). However, *post hoc* analysis of the Multicenter Randomized Clinical Trial of Endovascular Treatment for Acute Ischemic Stroke in the Netherlands (MR CLEAN) indicated that women had higher 90-day mortality and adverse events, compared to men (21). Whilst much of the analysis examining sex difference in EVT outcomes has relied on RCT data, real-world data from registries and surveys are more representative of the current clinical practice (19). Limited systematic reviews have been undertaken to summarize sex-specific utilization of EVT in routine practice. The aim of this systematic review was to examine sex differences in the utilization of EVT and clinical outcomes in patients with AIS from LVO.

## Methods

This systematic review was conducted according to the Preferred Reporting Items for Systematic Reviews and Meta-Analysis (PRISMA) guidelines (22), and the protocol was registered in international database (PROSPERO) of prospectively registered systematic reviews in health and social care (ID: CRD42021226100).

### Eligibility criteria and search

We searched PubMed/Medline and EMBASE for relevant articles published until December 2021. We used a combination of the following MeSH terms and keywords and their synonyms: stroke, brain ischemia, ischemic stroke, therapy, fibrinolytic agents, adverse effects, thrombectomy, mortality, thrombectomy trends, tissue plasminogen activator and female, male, sex characteristics, sex factors and sex difference. Details of the search strategy used in PubMed are given in Supplementary Appendix S1. The reference lists of any systematic or narrative reviews identified in the search and included studies were also screened for additional potentially relevant studies. We limited the search to studies undertaken in human adults.

Studies were included if they reported on adult women and men who received EVT for AIS, where EVT was defined as the intra-arterial use of a microcatheter, stent or other device for mechanical thrombectomy, with or without the use of a chemical thrombolytic agent (intra-arterial thrombolysis), and had a comparison group who received best medical management according to national and international guidelines, which may include intravenous thrombolysis. We also included studies which estimated sex differences in outcomes of EVT [such as functional outcome at discharge or after 90 days, mortality, symptomatic intracerebral hemorrhage (sICH), quality of life etc.]. Studies were excluded if they only included single-sex populations, did not focus on the treatment of EVT but explored the predictors, time onset, blood pressure indexes or procedure, were general discussions, contained duplicate data to another paper, were protocols or abstracts with no detailed data, case reports, or included fewer than 50 participants.

### Study selection and data extraction

Authors MO, SS, and XL scrutinized titles and abstracts, and excluded clearly irrelevant references independently. They independently reviewed abstracts of potential relevance to identify studies for review in full text. Two reviewers extracted data independently from the included studies. Where possible, the following data at study baseline were extracted from each report: year, first author and country of study, study size, number of women and men, age (years), the average length of follow-up (years), sex-stratified number of patients who received EVT or other management, study design, such as RCT or observational studies, such as national registries, hospital based or administrative data-based studies to make comparison with the study on use of thrombolysis (17). Hospital settings could include individual community-based or stroke-specialist centers or hospital networks. Administrative data included studies that used hospital billing data, such as hospital discharge data or Medicare. Any sex-stratified data on the following outcomes were also included: functional outcome defined by modified Rankin scale (mRS) at 90 days, mortality, sICH, and length of hospital stay measured using any measure. Any discrepancies in study selection or data extraction were resolved by mutual consent amongst the 3 authors.

### Quality assessment

For those studies examining sex differences in utilization of EVT, a modified quality assessment tool was developed by adapting items from Newcastle-Ottawa Scale (NOS) (23) and a recent systematic review of sex differences in intravenous thrombolysis treatment (17). Eligible studies were assigned a score of 0, 1, or 2 on the following four criteria: (1) the representativeness of the overall study population, (2) the proportion and impact of exclusions applied to the initial patient cohort, (3) adjustment of confounding variables, (4) the method by which the outcome (EVT or not) was ascertained. The overall study quality score will thus be in the range 0–8, where higher scores represent better study quality. A detailed description of the adapted quality assessment tool is provided in Supplementary Appendix S2. For studies which reported data on outcomes, the NOS scale was used for non-RCTs whilst the Grading of Recommendations, Assessment, Development and Evaluations (GRADE) tool was used for RCTs to determine the quality of evidence.

### Statistical analysis

Crude odds ratios (OR) for EVT, for women vs. men, were pooled across identified studies using random effects inverse-variance weighted meta-analysis. The adjusted OR was only reported in a single study. Subgroup analyses for observational studies were conducted by study design (hospital-based, registries or administrative), time of study publication (before 2015 or later), and geographic region (North America, South America, Europe, Asia or multiple regions). Similarly, subgroup analyses were also conducted for RCTs by publication year and geographic region.For those studies with multiple-adjusted sex-specific ORs for functional outcomes, EVT vs. not, were used to obtain women:men ratios (taking the maximum adjustment set) of ORs (RORs) (24). These were then pooled using the same method of meta-analysis as above. We used the *Q and I*^2^ statistics to assess heterogeneity, and contour-enhanced funnel plots were used to test for publication bias with 1%, 5% and 10% significance contours, and the regression-based Egger test was used to examine funnel-plot asymmetry for publication bias. Two-sided *p* values of less than or equal to 0.05 were deemed statistically significant. Meta-analyses used the *meta forestplot* command in Stata version 17 (25).

## Results

### Characteristics of included studies

**Figure 1:**
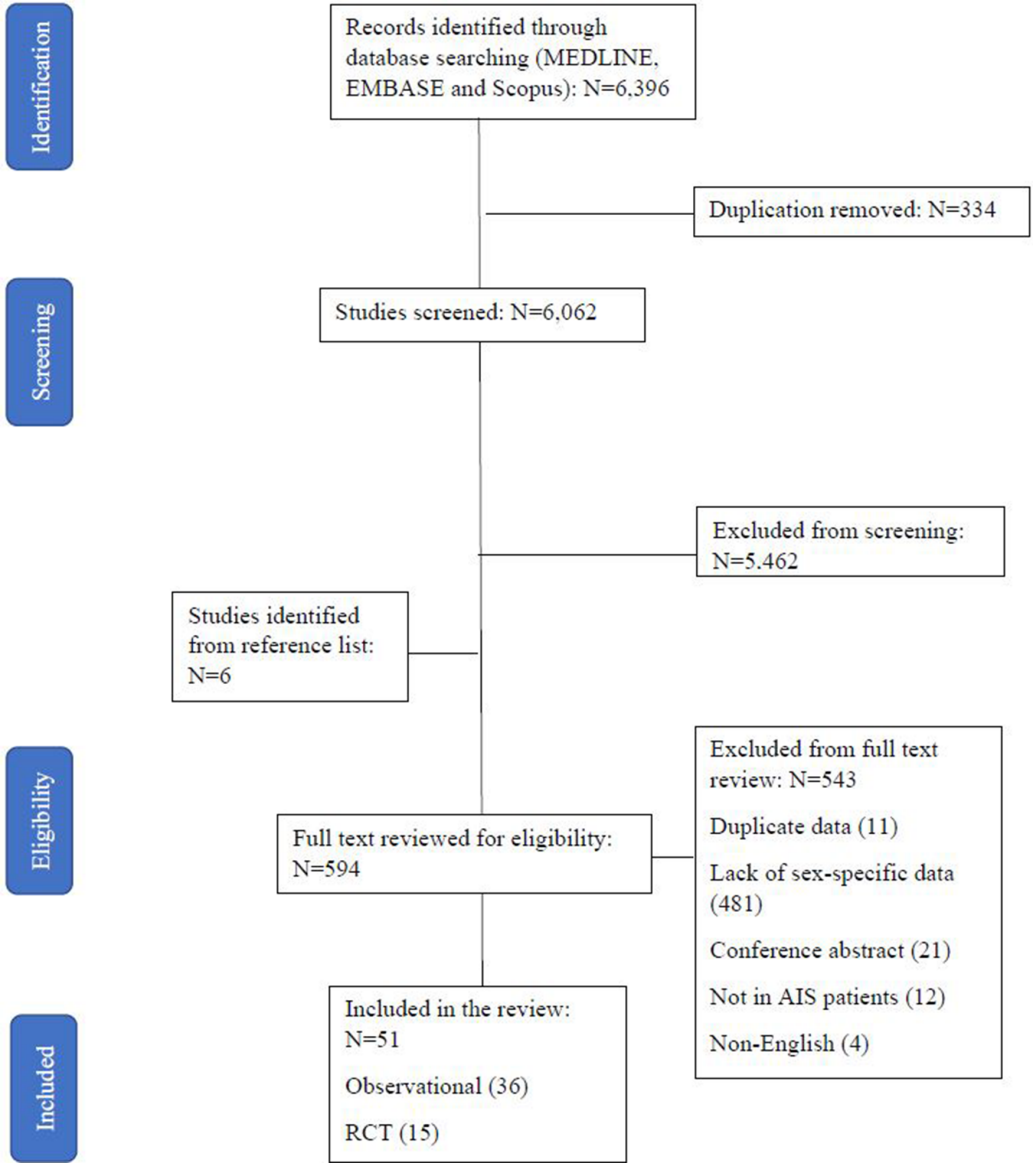
PRISMA flow diagram including reasons for exclusion of full-text articles.

Screening identified 6,396 studies, of which 594 were reviewed in full text (Figure 1). A total of 51 studies (26–72) (Supplementary Table S1) including 4,316,668 AIS patients (49.1% female) reported utilization of EVT stratified by sex in AIS patients with LVO. Nine of the studies (10, 11, 22, 29, 30, 36, 43, 57, 62) reported sex differences in EVT outcomes of mRS score at 90 days and two reported sex difference in mortality and safety outcome. Of all the included studies on EVT use, there were 36 observational studies (percentage of EVT use: 1.7%–78.0%) and 15 RCTs (33.3%–66.2%). The quality assessment score ranged from 2 to 6, with most of the studies scoring low to moderate quality scores (63% with a score of 3 to 4) (Supplementary Tables S2, S3).

### Sex-specified EVT use

**Figure 2:**
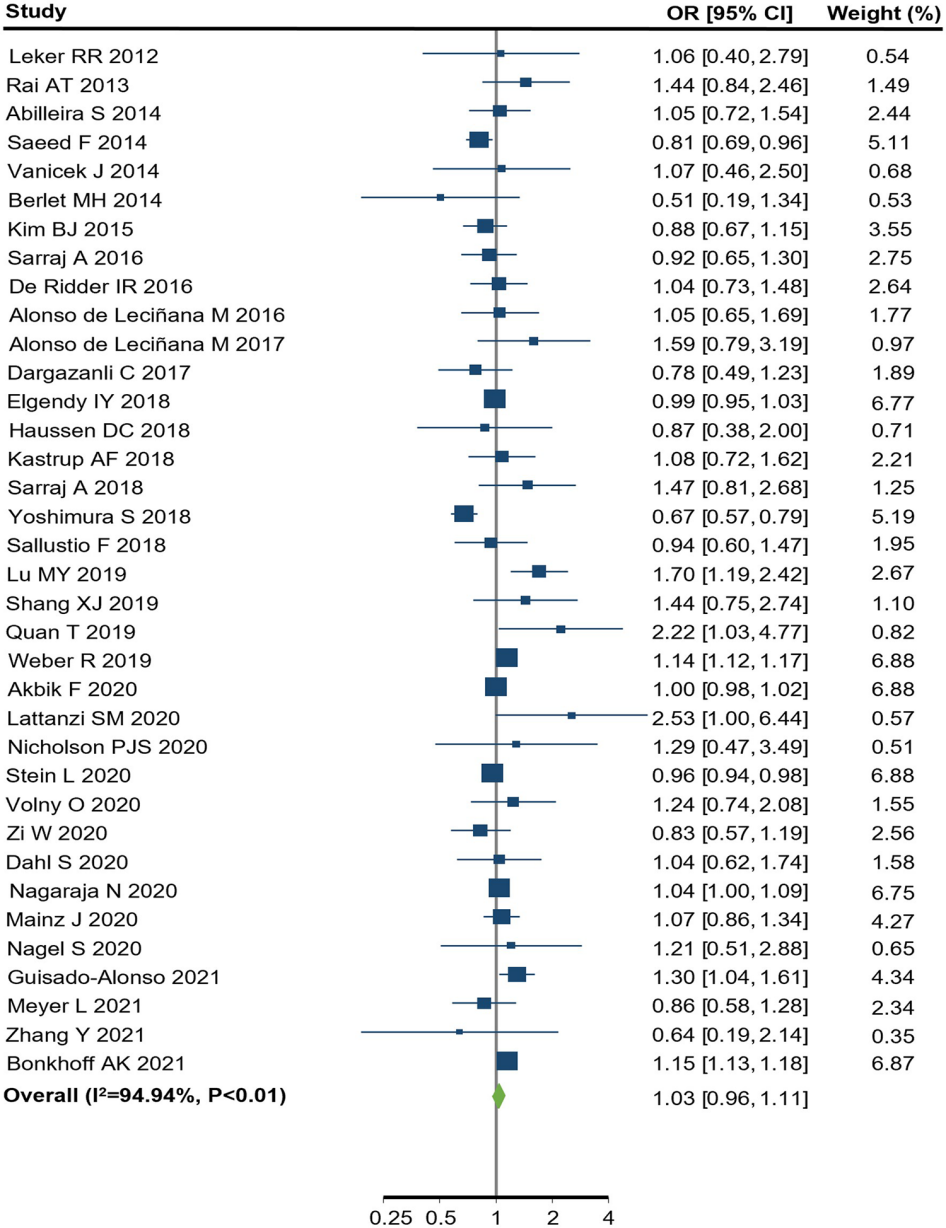
endovascular treatment use for women compared to men in observational studies. CI, confidence interval; OR, odds ratio. *p* value from Q statistics.

**Figure 3:**
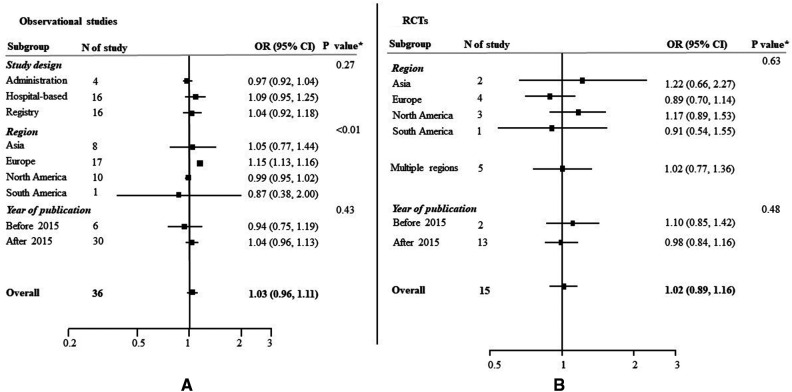
Odds ratios (OR) of EVT use for women compared to men, with 95% confidence intervals (CIs), by study type. CI, confidence interval; OR, odds ration; RCT, randomized controlled trial. *p value from Qb statistics.

Among the 36 observational studies which reported EVT use in women compared to men, the pooled crude OR was 1.03 (95% CI: 0.96–1.11) (Figure 2), indicating no significant sex difference in the odds of receiving EVT between women and men. The one study which reported an adjusted OR of 1.20, (95% CI: 0.92–1.56) for EVT use in women compared to men, adjusted for age, onset-to-door time and severity score (66). There was substantial heterogeneity among these observational studies, where the Q statistic was highly statistically significant (*p* < 0.01) and *I*^2^ was 95.0% (Figure 2). Figure 3 summarized the subgroup analysis of pooled estimates by region, publication year and study design. The findings of the pre-specified subgroup analysis by study design of observational studies were consistent with the main result: there was no significant difference of EVT use in women compared to men (registry OR: 1.04, 95% CI: 0.92–1.18; administrative data OR: 1.09, 95% CI: 0.95–1.25; hospital-based studies OR: 0.97, 95% CI: 0.92–1.04; test of group differences *p* = 0.27; Figure 3A, Supplementary Figure S1). Similar results were found in the subgroup analyses by publication year (Supplementary Figure S2). However, significant differerences were identified across region (*p* < 0.01); the studies conducted in Europe showed a higher odds of EVT use in women compared to men (OR: 1.15, 95% CI: 1.13–1.16; Supplementary Figure S3).

Similar results were found in the 15 included RCTs, with no evidence of substantial heterogeneity (OR: 1.02, 95% CI: 0.89–1.16; *I*^2 ^= 0.0%, *p* = 0.68; Figure 3B; Supplementary Figure S4). No heterogeneity was identified across the subgroup analysis by publication year (Supplementary Figure S5) and region (Supplementary Figure S6).

### Sex differences in clinical outcomes of EVT

**Figure 4:**
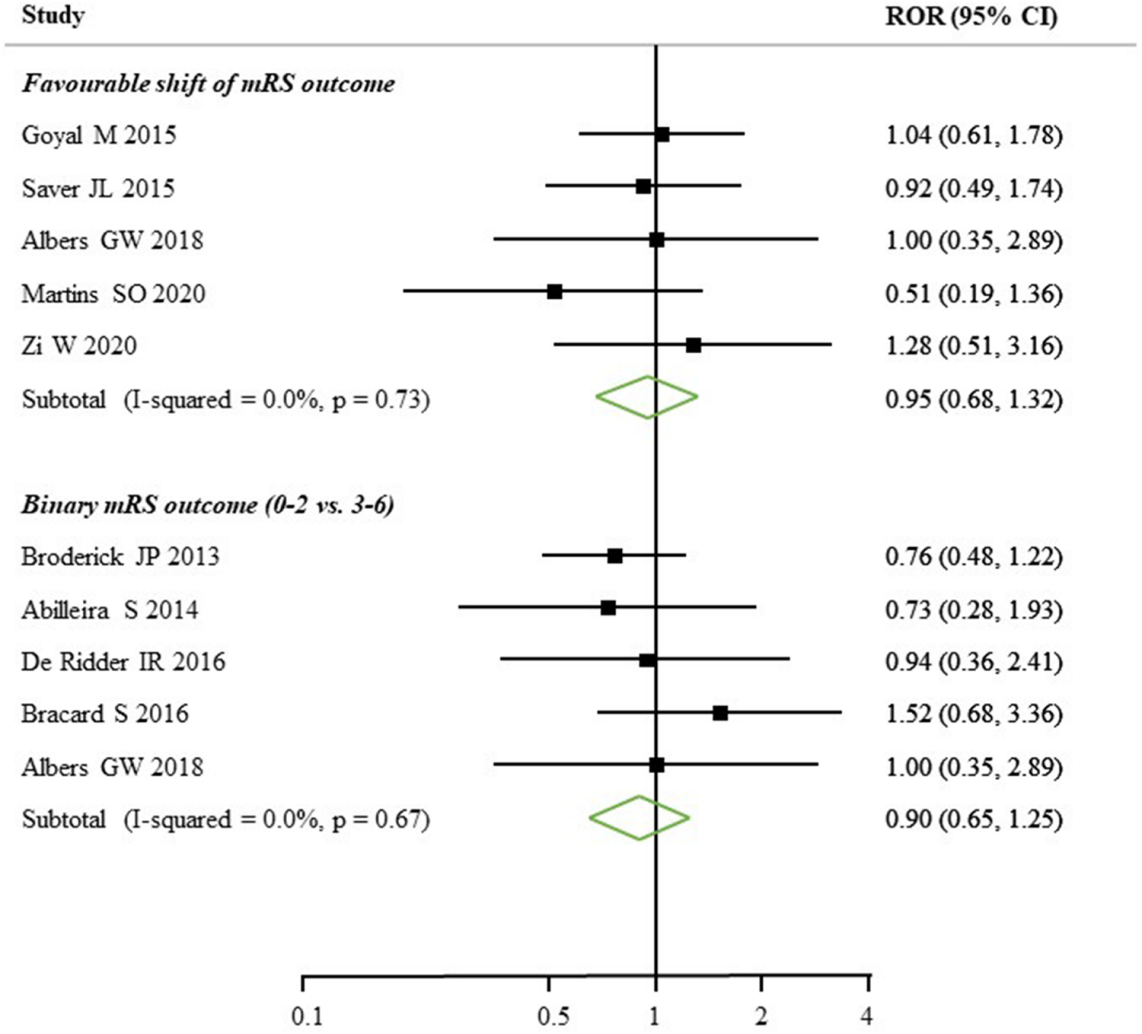
Women to men ratios of sex-specific odds ratios, with 95% confidence intervals (CIs), of functional outcomes after EVT treatment. mRS, modified Rankin scale; ROR, relative ratio of odds ratios.

Only five (4 RCTs and 1 Registry-based study) out of the 52 studies reported sex-specific estimates of clinical outcomes on favourable shift of mRS (Figure 4A). There was no significant difference found on favourable shift of mRS for women relative to men (ROR: 0.95, 95% CI: 0.68–1.32) and no heterogeneity identified across the studies (*I*^2 ^= 0.0%, *p* = 0.73). Of another five studies (3 RCTs and 2 Registry-based studies), the women to men ROR for reported functional outcome, by mRS score 0–2 vs. 3–6, was 0.90 (95% CI: 0.65–1.25), with no heterogeneity identified (*I*^2 ^= 0.0%, *p* = 0.67, Figure 4B). Only two studies reported sex differences in death or safety outcomes, such as sICH. There was a higher percentage of in-hospital mortality in women (16.32% vs. 12.60%) and sICH (9.2% vs. 6.7%) compared to men (Supplementary Table S4).

### Publication bias

The funnel plots were almost symmetrical, with no significant publication bias observed through the Egger regression-based test in the results that focused on utilization of EVT (Supplementary Figure S7) and EVT outcomes (Supplementary Figure S8).

## Discussion

This review provides a comprehensive analysis of the current evidence on sex differences in the utilization and outcomes of EVT. Among 4,316,668 AIS patients, in 51 studies, we found no significant difference in the use of EVT among women compared to men, either in observational studies or RCTs. Furthermore, there was no sex difference in the functional outcomes of EVT according to the mRS score assessed at 90 days.

A recent meta-analysis showed that women were less likely than men to receive intravenous thrombolysis (17). Similarly, a study that examined data available from 1997 to 2006 showed that women were also less likely to be given several revascularisation interventions after stroke (73). However, our review demonstrated that sex did not modify the utilization of EVT, which might be related to an increased uptake of reperfusion treatment over time. This is consistent with recent evidence highlighting an increase in the utilization of EVT in both sexes when data were restricted to the period, 2006 to 2014 (74). Our subgroup analysis also supports this result, which showed that before 2014, the utilization of EVT was significantly lower in women. However, in subgroup analysis by region, there was significant differences between the groups. Women were more likely to receive EVT compared to men in studies conducted in Europe. This might be explained by the greater weight given by a European registry on EVT, which reflects the high capacity of EVT treatment in this region as 40/44 European countries were reported providing endovascular procedures to patients with AIS (75). A study conducted in UK has shown that women had a higher frequency of anterior circulation LVO AIS than men (76), and which were more likely to be eligible to undertake EVT according to clinical guideline recommendations. Women experience delayed arrival to hospital and door-to-scan times due to differences in acute stroke presentation compared to men (77, 78). Therefore, they were ineligible for thrombolysis and more likely to receive EVT with a prolonged time window. Though the pooled estimates shows no sex differences on EVT use from Asia, there was significant differences of EVT utilisation across the studies within the region group. This may be due to accessibility of EVT, adequacy of specialist available to perform procedures and availability of stroke/neuro-interventional units differed across countries in Asia (79).

Our findings were consistent to a previously published meta-analyses restricted to RCTs which found that sex does not modify functional outcomes after EVT; concluding that the treatment should be considered equally for women and men (21). However, their results were based exclusively on RCT data, and did not allow an assessment of differences in real-world circumstances. As RCTs are designed with particular inclusion and exclusion criteria for the purposes of efficiently assessing outcomes for a particular outcome, they may not be fully representative of a diverse range of real-world populations (80). Although there is a recent published study of multi-national registries which has shown no sex differences on the functional outcomes after EVT conducted in the late time window (6–24 h), the observational studies on the sex disparities on EVT treatment are still scarce (81). It is, therefore, important to include any administrative or registry based data for such analysis to determine if there is any significant modification emerging in the association of sex and outcomes after EVT in clinical practice.

### Implications for clinical practice and future research

From our review, we can conclude that in real-world data, such as registry-based, hospital-based or administrative data, there are no sex differences in the utilization and outcomes of EVT in AIS. Our findings reflect pooled estimates of a previously published meta-analysis on RCTs (21), that is, of no sex differences in EVT outcomes, and therefore both women and men should be considered for EVT where possible.

However, future research in this area should consider reporting data by sex to determine any potential sex differences in outcomes after EVT. Only five studies provided sex stratified data on functional outcomes after EVT. The majority of the RCTs (6/10) did not report functional outcomes by sex, and even fewer real-world studies reported sex-specified outcomes. This will be crucial in determining if real world outcomes are different compared to RCTs. According to a recent statement from the American Heart Association/American Stroke Association, evidence is still lacking regarding sex differences in long-term outcomes after EVT (6). Further evaluation of additional outcome measures, such as long-term disability, health-related quality of life, and post-stroke depression and other social domains, is essential to have a thorough understanding of sex differences in EVT outcomes (6).

### Strengths and limitations

This study had several strengths. We utilized a broad search strategy to identify all relevant studies, and duplicate screening and extraction was conducted to reduce reviewer bias. We also conducted subgroup analysis by publication year and region to explore any potential heterogeneity. We also included hospital-based, administrative and registry data to reduce selection bias that can be induced by only including RCTs. However, there were also some limitations. Our analysis was based on crude estimates of EVT utilization, since limited studies reported adjusted estimates, and most of the studies were identified as having low to moderate level quality based on the quality assessment. In addition, the esitmates of sex differences in EVT utilization in RCTs were due to random chance.

## Conclusion

There were no sex differences in the utilization and functional outcomes of EVT for patients with AIS. However, further research is needed using real-world data to identify long-term sex-stratified outcomes after EVT.

## Data Availability

The raw data supporting the conclusions of this article will be made available by the authors, without undue reservation.
