# Supplementary material for: Sex differences in the utilization and outcomes of endovascular treatment after acute ischemic stroke: A systematic review and meta-analysis
Source: Front Glob Womens Health. 2023 Jan 18;3:1032592. doi: 10.3389/fgwh.2022.1032592 (PMC9889638; doi:10.3389/fgwh.2022.1032592)
Supplement: Supplementary file 1 [file Datasheet1.pdf]

Supplementary Material

Sex differences in the utilization and outcomes of endovascular thrombectomy after acute ischemic stroke: a meta-analysis and systematic review

Menglu Ouyang, et al.

Contents

Appendix 1. PubMed Search strategy 2

Appendix 2. Quality assessment 2

Table S1. Characteristics of included studies 3

Table S2: Quality Assessment of Treatment Rates using NOS 5

Table S3: Quality Assessment of Functional Outcomes 7

Table S4: Sex difference in mortality and safety outcome 8

Figure S1. Subgroup analysis by study design of observational studies 9

Figure S2. Subgroup analysis by study year of observational studies 10

Figure S3. Subgroup analysis by study region of observational studies 11

Figure S4. Endovascular treatment use for women compared to men in randomized controlled trials 12

Figure S5. Subgroup analysis by study year of randomized controlled trials 13

Figure S6. Subgroup analysis by region of randomized controlled trials 14

Figure S7. Funnel plot of publication bias in studies for utilisation of endovascular treatment compared women to men 15

Figure S8. Funnel plot of publication bias in studies for EVT outcomes 16

Appendix 1. PubMed Search strategy

(("stroke/drug therapy"[MeSH Terms] OR "stroke/surgery"[MeSH Terms] OR "stroke/therapy"[MeSH Terms] OR "brain ischemia/drug therapy"[MeSH Terms] OR "brain ischemia/surgery"[MeSH Terms] OR "brain ischemia/therapy"[MeSH Terms] OR "ischemic stroke/drug therapy"[MeSH Terms] OR "ischemic stroke/surgery"[MeSH Terms] OR "ischemic stroke/therapy"[MeSH Terms]) AND ("fibrinolytic agents/adverse effects"[MeSH Terms] OR "fibrinolytic agents/therapeutic use"[MeSH Terms] OR "thrombectomy/adverse effects"[MeSH Terms] OR "thrombectomy/therapeutic use"[MeSH Terms] OR "thrombectomy/therapy"[MeSH Terms] OR "tissue plasminogen activator/adverse effects"[MeSH Terms] OR "Endovascular Procedures/methods"[Mesh] OR "Endovascular Procedures/trends"[Mesh]) AND ("female"[MeSH Terms] OR "male"[MeSH Terms] OR "sex characteristics"[MeSH Terms] OR ("sex factors"[MeSH Terms] OR "f factor"[MeSH Terms]) OR "sex difference*"[Title/Abstract])) AND ((humans[Filter]) AND (alladult[Filter]))

Appendix 2. Quality assessment

Studies were assessed based on the representativeness of the overall study population, the number and impact of exclusions applied to the initial patient cohort, adjustment for potential confounding variables that affect endovascular thrombectomy treatment, and the method by which the outcome (endovascular thrombectomy treatment or not) was ascertained. With regard to representativeness, a score of *0* was given if the study included data from only one or two hospitals, *1* if the study was regional in scope and included multiple hospitals or studied a specific subset of hospitals (i.e. academic medical centers), and *2* if it was national in scope. For the number and impact of exclusions, a score of *0* was given if there were significant exclusions (>15%) from the starting cohort; *1* if the subjects under study comprised a random sample of a consecutive cohort, were taken only from stroke units, or represented cases selected as part of a randomized clinical trial; and *2* if it was a "gold standard" study (i.e. consecutive cohort with <15% exclusions). Regarding adjustment for confounding variables, a score of *0* was awarded for no adjustment, *1* for any adjustment at all, and *2* for full adjustment (e.g. age, severity, comorbidities, pre-stroke function, etc.). Finally, for the method by which the outcome was ascertained, a score of *0* was given if there was no documentation of how the outcome was ascertained, *1* if it was done through billing codes, and *2* if it was done through the medical record.

Reference: Strong, Brent, Lynda D. Lisabeth, and Mathew Reeves. "Sex differences in IV thrombolysis treatment for acute ischemic stroke: a systematic review and meta-analysis." *Neurology* 95, no. 1 (2020): e11-e22. https://doi.org/10.1212/WNL.0000000000009733

Table S1. Characteristics of included studies

Ref ID	Year of study	Author	Region	Study design	No. participants	No. of EVT men treated	No. of EVT women treated	total No. of men	total No. of women
**16**	2012	Leker RR, et al.	Asian	Registry	88	11	11	45	43
**17**	2013	Ciccone A, et al.	Europe	RCT	362	106	75	209	153
**18**	2013	Rai AT, et al.	North America	Hospital-based	223	64	59	125	98
**19**	2013	Broderick JP, et al.	North America	RCT	656	218	216	340	316
**20**	2014	Abilleira S, et al.	Europe	Registry	1179	64	55	647	532
**21**	2014	Saeed F, et al.	North America	Administration	2313	471	444	1119	1194
**22**	2014	Vanicek J, et al.	Europe	Hospital-based	87	25	21	48	39
**23**	2014	Berlet MH, et al.	North America	Hospital-based	76	15	10	37	39
**24**	2015	Goyal M, et al.	Multicentre	RCT	315	79	86	150	165
**25**	2015	Campbell BCV, et al.	Multicentre	RCT	70	17	17	35	35
**26**	2015	Saver JL, et al.	Multicentre	RCT	196	54	44	98	98
**27**	2015	Jovin TG, et al.	Multicentre	RCT	206	55	48	109	97
**28**	2015	Kim BJ, et al.	Asia	Registry	872	184	155	456	416
**29**	2016	Bracard S, et al.	Multicentre	RCT	402	115	85	217	185
**30**	2016	Sarraj A, et al.	North America	Hospital-based	522	144	144	256	266
**31**	2016	De Ridder IR, et al.	Europe	Hospital-based	500	135	98	292	208
**32**	2016	Alonso de Leciñana M, et al.	Europe	Hospital-based	303	106	95	161	142
**33**	2017	Muir KW, et al.	Multicentre	RCT	65	13	20	29	36
**34**	2017	Alonso de Leciñana M, et al.	Europe	Hospital-based	131	33	41	65	66
**35**	2017	Dargazanli C, et al.	Europe	Hospital-based	301	95	75	160	141
**36**	2017	Khoury NN, et al.	North America	RCT	77	18	22	38	39
**37**	2018	Albers GW, et al.	Multicentre	RCT	182	46	46	90	92
**38**	2018	Elgendy IY, et al.	North America	Administration	70046	5464	5331	35255	34791
**39**	2018	Haussen DC, et al.	South America	Hospital-based	118	15	15	56	62

**40**	2018	Kastrup AF, et al.	Europe	Hospital-based	426	64	145	134	292
**41**	2018	Nogueira RG, et al.	Multicentre	RCT	206	42	65	93	113
**42**	2018	Sarraj A, et al.	North America	Hospital-based	200	72	52	123	77
**43**	2018	Yoshimura S, et al.	Asian	Registry	2399	757	521	1,312	1,087
**44**	2018	Sallustio F, et al.	Europe	Registry	325	58	74	140	185
**45**	2018	Zhao QS, et al.	Asian	RCT	90	21	9	62	28
**46**	2019	Lu MY, et al.	Asian	Registry	2813	62	67	1703	1110
**47**	2019	Shang XJ, et al.	Asian	Hospital-based	177	52	27	124	53
**48**	2019	Quan T, et al.	Asian	Hospital-based	159	61	28	119	40
**49**	2019	Weber R, et al.	Europe	Registry	1112570	20407	21995	571401	541169
**50**	2020	Akbik F, et al.	North America	Registry	267956	21479	21651	133585	134371
**51**	2020	Martins SO, et al.	Multicentre	RCT	221	60	51	117	104
**52**	2020	Lattanzi SM, et al.	Europe	Registry	88	28	22	57	31
**53**	2020	Nicholson PJS, et al.	North America	Hospital-based	66	14	12	38	28
**54**	2020	Stein L, et al.	North America	Hospital-based	1782710	17433	17413	873501	909209
**55**	2020	Volny O, et al.	Europe	Hospital-based	236	60	79	107	129
**56**	2020	Zi W, et al.	Asian	Registry	829	483	164	612	217
**57**	2020	Dahl S, et al.	Europe	Registry	1226	32	29	655	571
**58**	2020	Nagaraja N, et al.	North America	Administration	468630	3855	4125	231120	237510
**59**	2020	Liu X, et al.	Asia	RCT	131	48	18	100	31
**60**	2020	Mainz J, et al.	Europe	Registry	5356	188	152	3048	2308
**61**	2020	Nagel S, et al.	Europe	Registry	94	15	15	50	44
**62**	2021	Guisado-Alonso, et al.	Europe	Registry	2203	203	202	1220	983
**63**	2021	Meyer L, et al.	Europe	Registry	432	99	69	245	187
**64**	2021	Zhang Y, et al.	Asia	Hospital-based	58	14	5	39	19
**65**	2021	Langezaal LCM, et al.	Multinational	RCT	300	100	54	196	104
**66**	2021	Bonkhoff AK, et al.	Europe	Registry	587661	16448	14101	335857	251804

RCT denotes randomized controlled trial

Table S2: Quality Assessment of Treatment Rates using NOS

Study ID	Representativeness	Exclusion	Adjustment	Outcome	Overall NOS score
**A. Non-RCTs**					
**Abilleira S, et al. 2014**	2	0	0	2	4
**Akbik F, et al. 2020**	2	0	0	2	4
**Elgendy IY, et al. 2018**	2	0	2	2	6
**Alonso de Leciñana M, et al. 2017**	1	0	0	2	3
**Dargazanli C, et al. 2017**	2	0	0	0	2
**Haussen DC, et al. 2018.**	0	0	0	2	2
**Kastrup AF, et al. 2018**	0	0	0	2	2
**Lu MY, et al. 2019**	0	2	0	2	4
**Lattanzi SM, et al. 2020**	0	0	0	2	2
**Leker RR, et al. 2012**	0	0	0	2	2
**Nicholson PJS, et al. 2020**	0	0	0	2	2
**Saeed F, et al. 2014**	2	0	0	2	4
**Sarraj A, et al. 2018**	1	0	0	2	3
**Sarraj A, et al. 2016**	1	0	0	2	3
**Shang XJ, et al. 2019**	1	1	0	2	4
**Stein L, et al. 2020**	2	0	0	2	4
**Vanicek J, et al. 2014**	0	0	0	2	2
**Volny O, et al. 2020**	1	0	0	2	3
**Yoshimura S, et al. 2018**	2	2	0	2	6
**Zi W, et al. 2020**	2	0	0	2	4
**Quan T, et al. 2019**	1	0	0	2	3
**Sallustio F, et al. 2018**	0	0	0	2	2
**De Ridder IR, et al. 2016**	0	2	0	2	4
**Dahl S, et al. 2020**	1	0	0	2	3
**Nagaraja N, et al. 2020**	2	2	0	2	6
**Weber R, et al. 2019**	2	2	0	2	6
**Meyer L, et al. 2021**	2	0	0	2	4
**Zhang Y, et al. 2021**	0	0	0	2	2
**Kim BJ, et al. 2015**	2	0	0	2	4
**Mainz J, et al. 2020**	2	2	0	2	6
**Nagel S, et al. 2020**	0	0	0	2	2
**Alonso de Leciñana M, et al. 2016**	2	0	0	2	4
**Rai, et al. 2013**	0	2	0	2	4
**Guisado-Alonso, et al. 2019**	2	0	0	2	4
**Berlet, M. H. et al. 2014**	0	0	0	2	2
**Bonkhoff AK. et al. 2021**	2	0	2	2	6

**B. RCTs**					
**Albers GW, et al. 2018**	0	2	0	2	4
**Bracard S, et al. 2016**	2	2	0	2	6
**Martins SO, et al. 2020**	0	2	0	2	4
**Nogueira RG, et al. 2018**	0	2	0	2	4
**Zhao, QS, et al. 2018**	0	2	0	2	2
**Goyal M, et al. 2015**	0	2	0	2	3
**Campbell BCV, et al. 2015**	0	2	0	1	3
**Saver JL, et al. 2015**	0	2	0	1	3
**Jovin TG, et al. 2015**	0	2	0	2	4
**Muir KW, et al. 2016**	0	2	0	2	4
**Khoury NN, et al. 2017**	0	2	0	2	4
**Langezaal LCM, et al. 2021**	0	2	0	2	4
**Liu X, et al. 2019**	0	2	0	1	3
**Ciccone, A. et al. 2013**	0	2	0	2	4
**Broderick, J. P. et al. 2013**	0	2	0	2	4

Table S3: Quality Assessment of Functional Outcomes

**Study ID**									
**A. Non-RCTs**	**Selection**				**Comparability**	**Outcome**			**Overall NOS score**
**Zi W, et al. 2020**	1	1	1	1	2	1	1	1	9
**Abilleira S, et al. 2014**	1	1	1	0	2	0	1	0	6
**deRidder IR, et al. 2016**	1	1	1	1	2	1	1	1	9
**B. RCTs**	**Risk of bias**	**Inconsistency of results**	**Indirectness of evidence**	**Imprecision**	**Publication bias**	**Large magnitude of effect**	**All condounfdng reduced**	**Dose response gradient**	**Quality of evidence grades**
**Albers GW, et al. 2018**	moderate	low	low	very low	high	large	very low	NA	low
**Bracard S, et al. 2016**	high	high	low	high	high	small	low	NA	moderate
**Martins SO et al. 2020**	high	high	low	very low	moderate	large	very low	NA	moderate
**Saver JL, et al. 2015**	moderate	low	low	very low	low	small	low	NA	moderate
**Goyal M, et al. 2015**	high	low	low	low	low	large	very low	NA	moderate
**Broderick, J. P. et al. 2013**	moderate	low	low	very low	low	large	very low	NA	low

Table S4: Sex difference in mortality and safety outcome

Ref ID	Author	Men	Women
**29**	**De Ridder IR, et al. 2016**		
	Death within 7 days (%)	17/135 (12.60%)	16/98 (16.32%)
	OR (95% CI) of death	2.19 (1.24-3.87)	2.05 (0.96-4.35)
	sICH (%)	9/135 (6.7%)	9/98 (9.2%)
**58**	**Nagaraja N, et al. 2020**		
	In-hospital death (%)	650/1355 (47.97%)	650/1685 (38.58%)
	Adjusted OR (95% CI) of death, Male:Female	EVT alone 1.01 (0.73-1.40)<br>t-PA + EVT 1.28 (0.67-2.43)	
	Prolonged hospital stay	1,490/ 5,175 (28.79%)	1,345/ 5,470 (24.59%)
	Adjusted OR (95% CI) of prolonged hospital stay, Male:Female	EVT alone 0.89 (0.70-1.15)<br>t-PA + EVT 0.71 (0.46-1.09)	

M=Men; W=Women; sICH=symptomatic intracerebral hemorrhage; LOS=Length of Stay.

Figure S1. Subgroup analysis by study design of observational studies

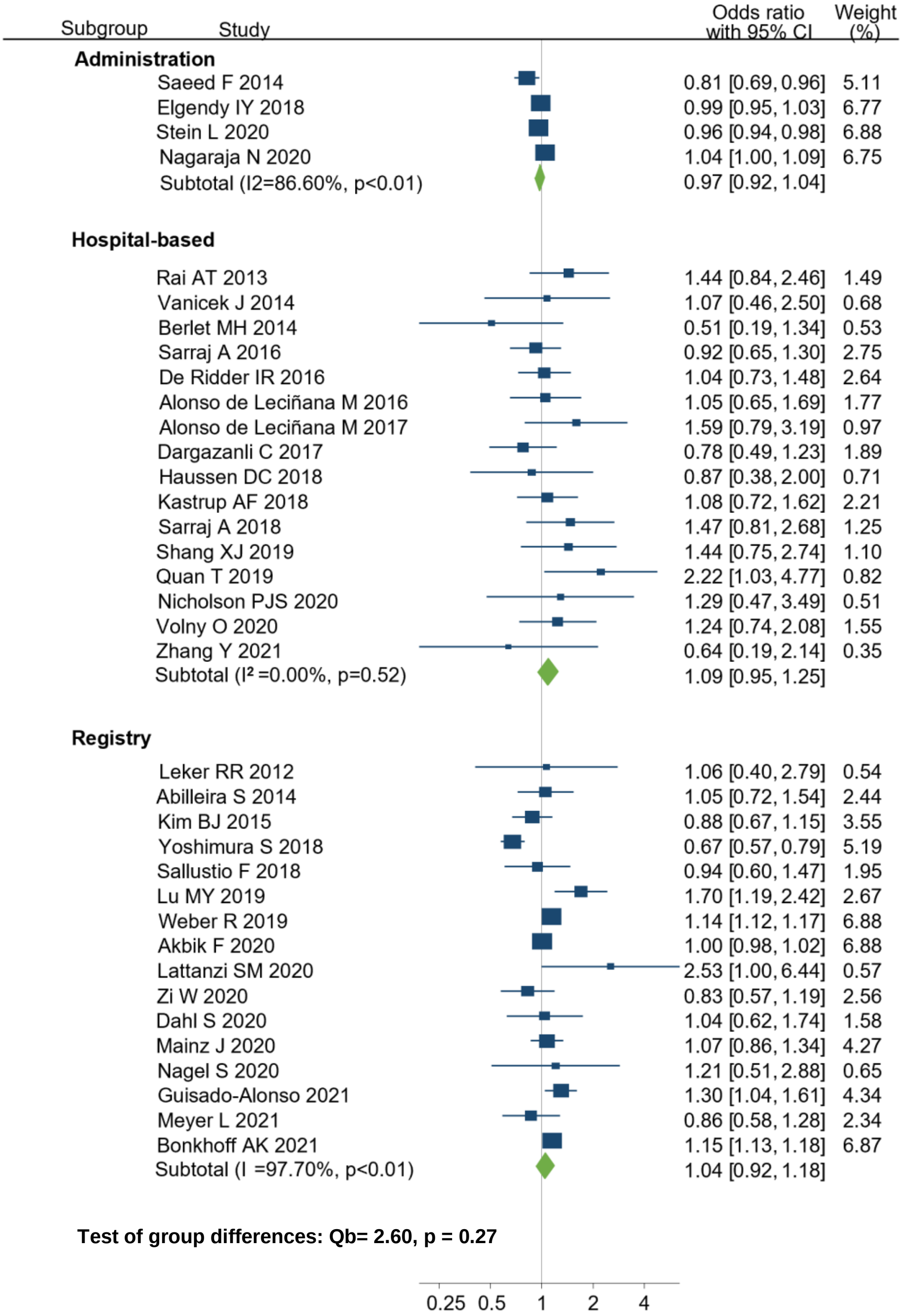

Footnote: CI denotes confidence interval

Figure S2. Subgroup analysis by study year of observational studies

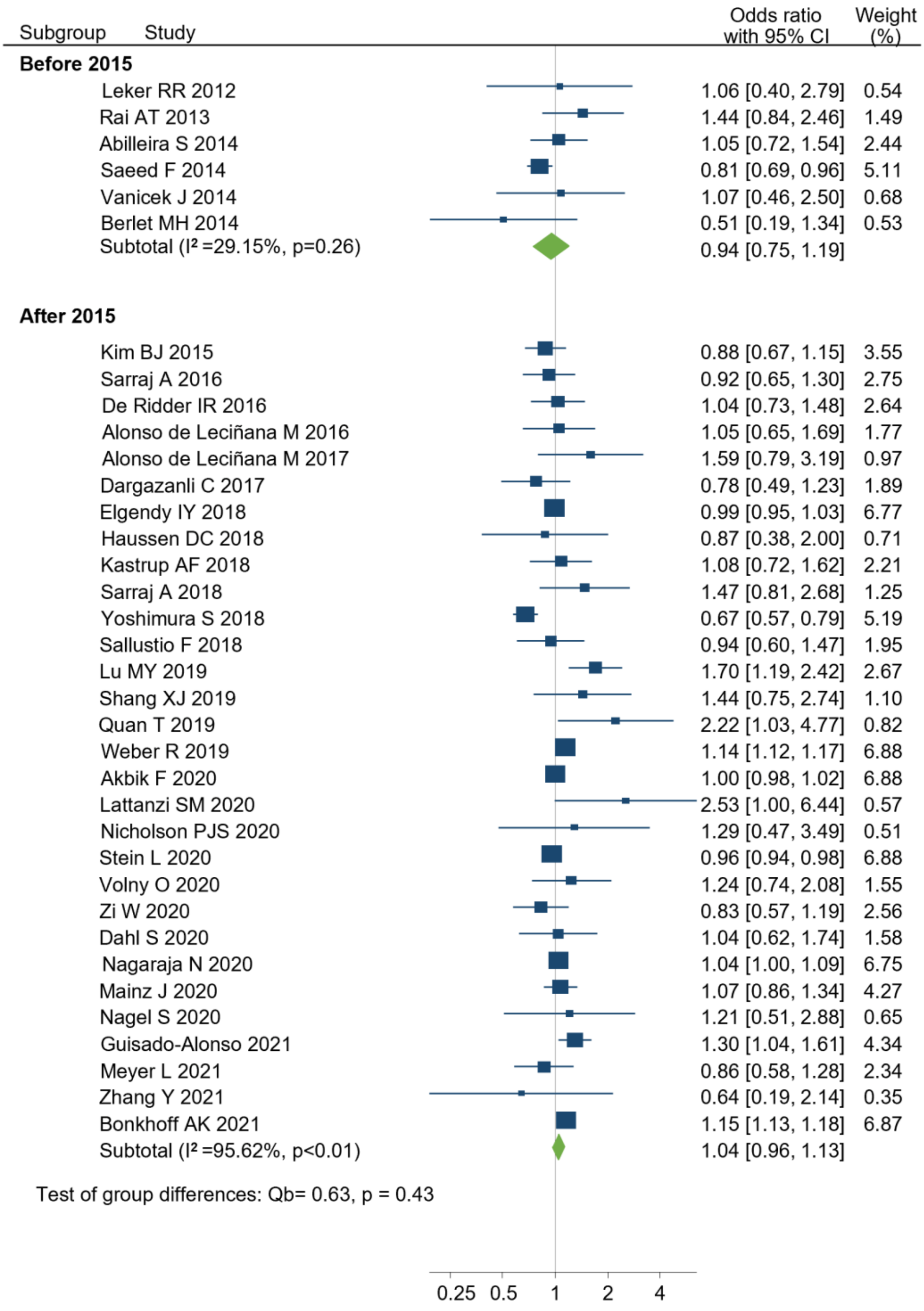

Footnote: CI denotes confidence interval

Figure S3. Subgroup analysis by study region of observational studies

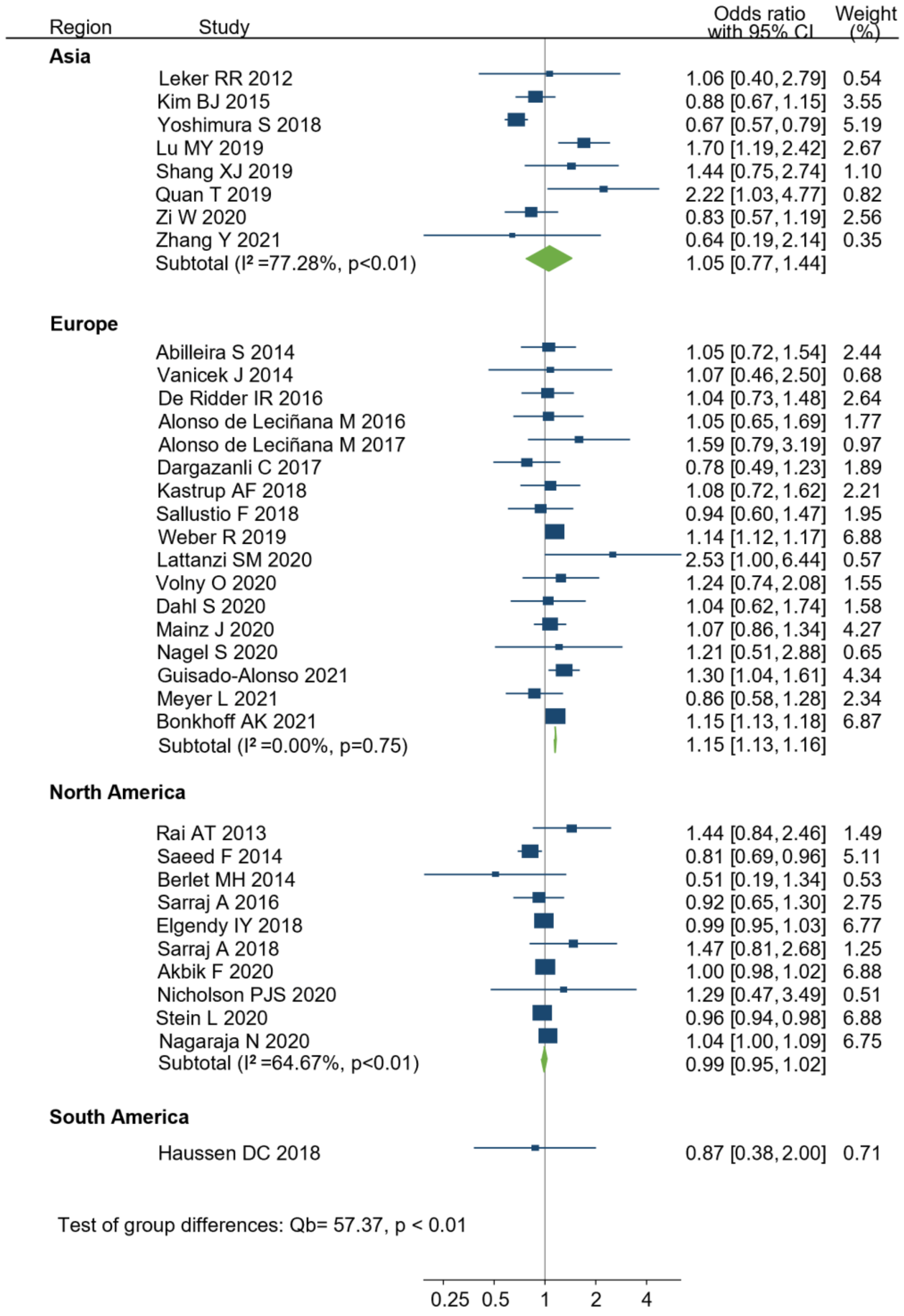

Footnote: CI denotes confidence interval

Figure S4. Endovascular treatment use for women compared to men in randomized controlled trials

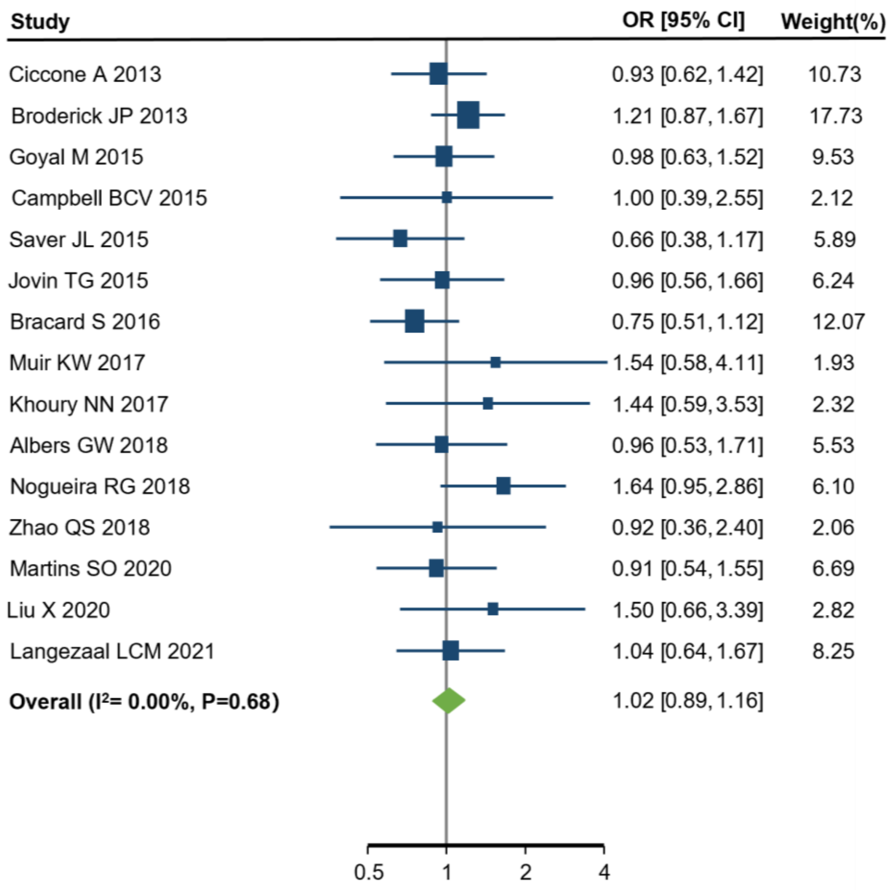

Footnote: CI denotes confidence interval, OR odds ration, RCT randomized controlled trial
P value from Q statistics

Figure S5. Subgroup analysis by study year of randomized controlled trials

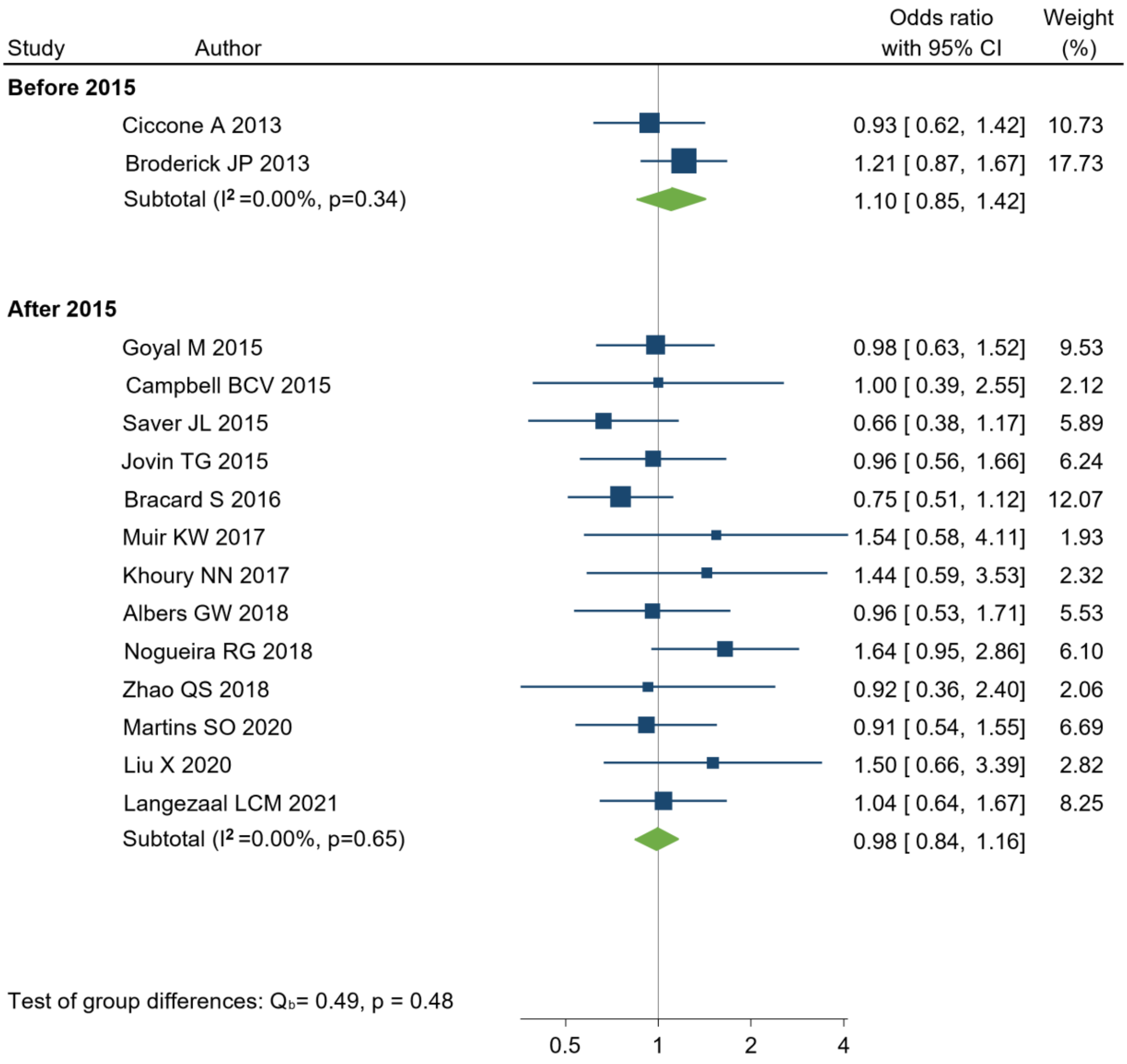

Footnote: CI denotes confidence interval

Figure S6. Subgroup analysis by region of randomized controlled trials

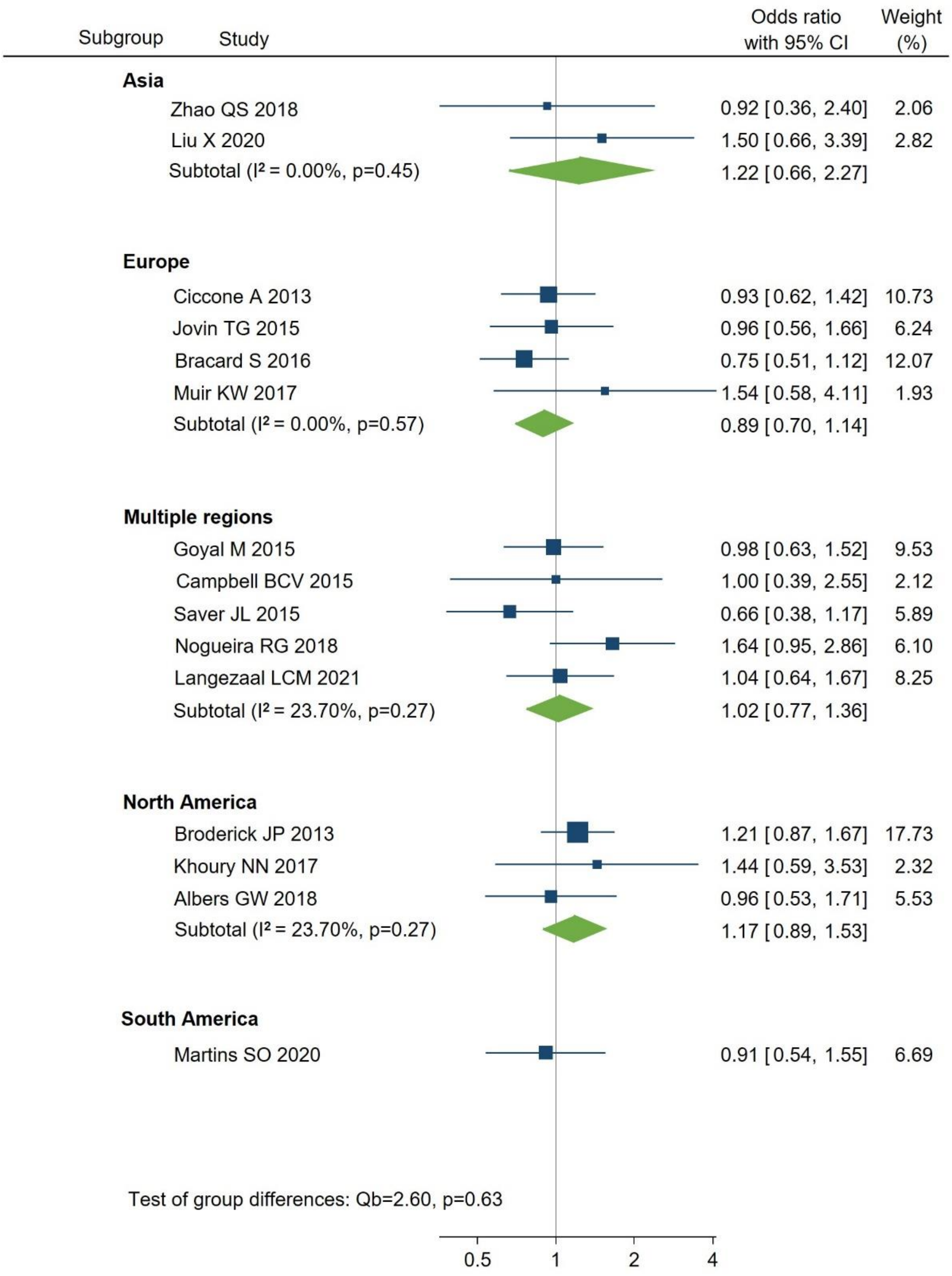

Footnote: CI denotes confidence interval

Figure S7. Funnel plot of publication bias in studies for utilisation of endovascular treatment compared women to men

A. Observational studies

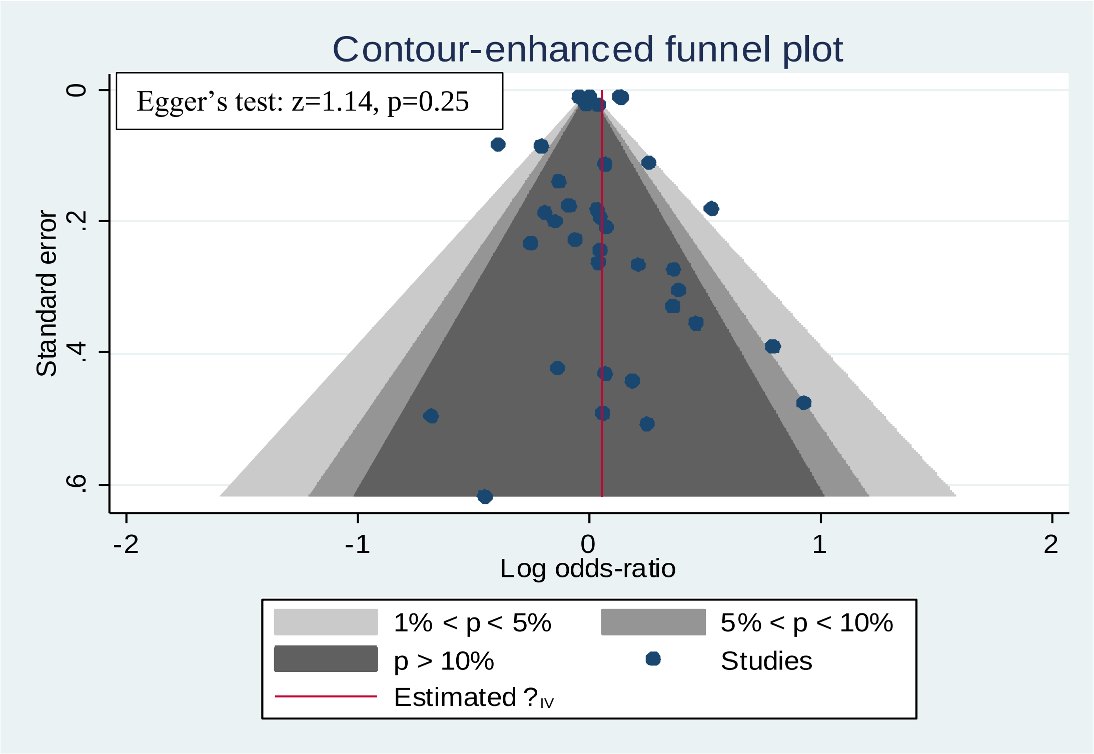

B. Randomized controlled trials

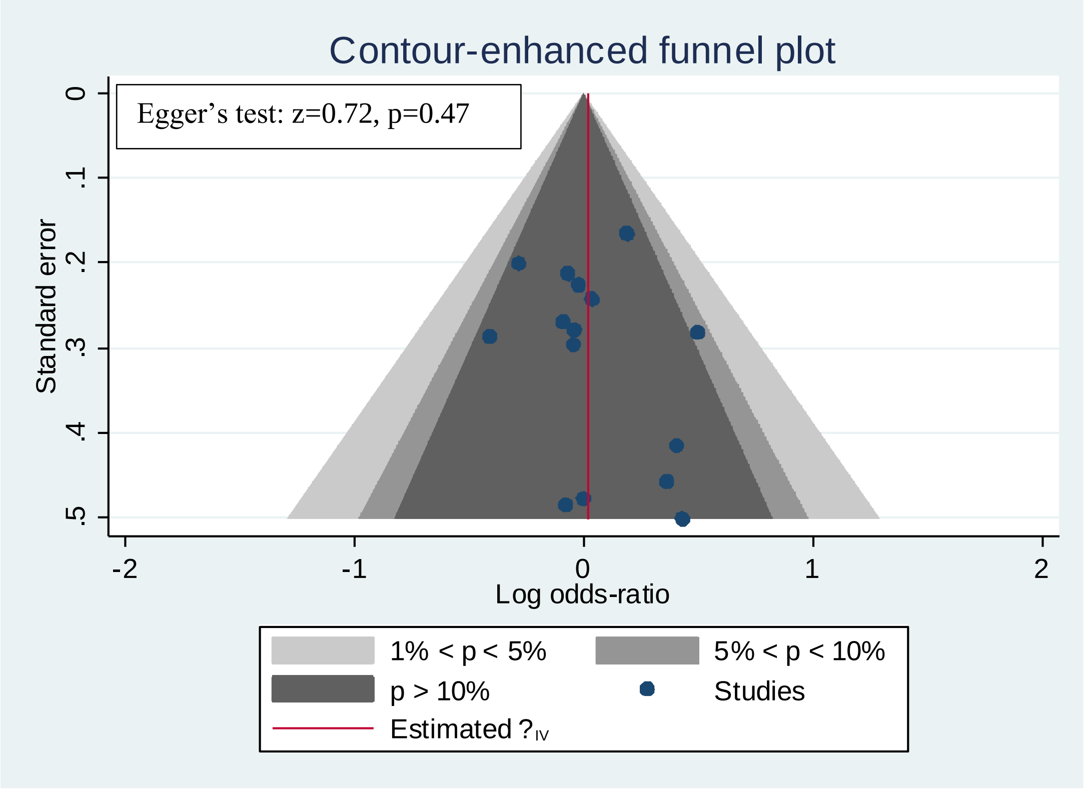

<u>Figure S8. Funnel plot of publication bias in studies for EVT outcomes</u>

A. Favourable shift of mRS outcome

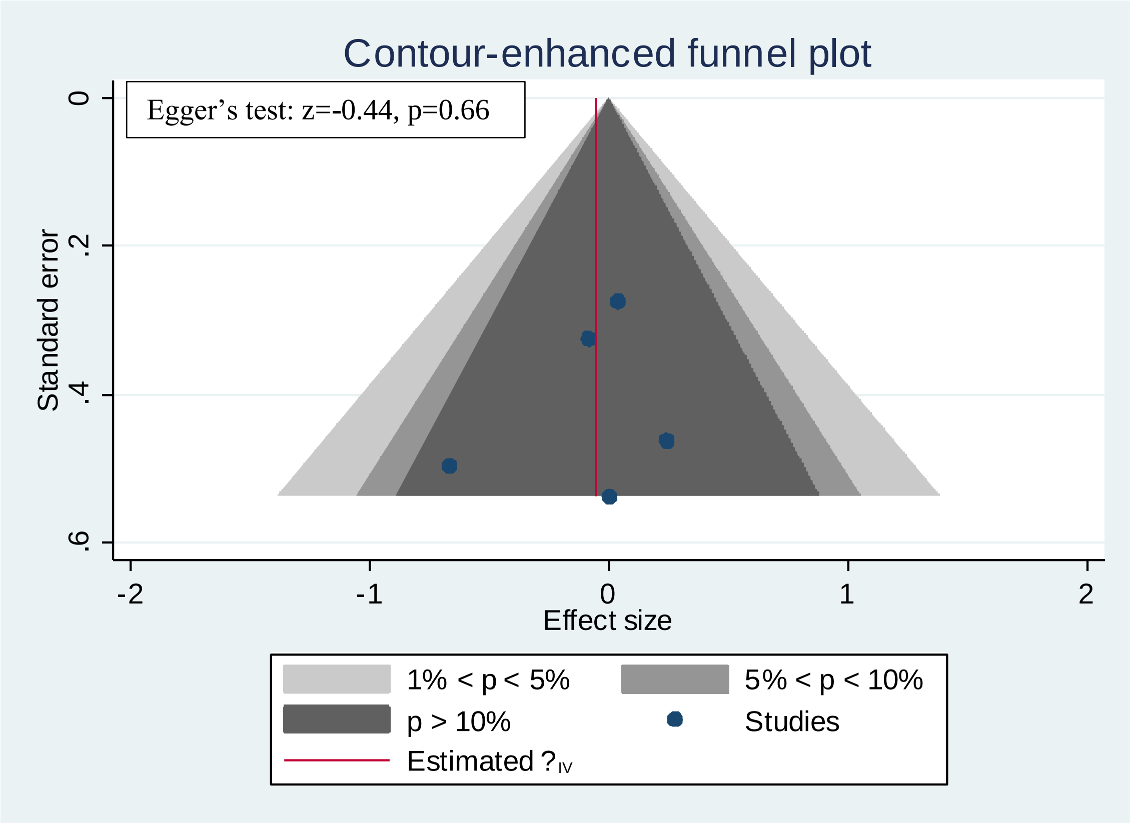

B. Binary mRS outcomes (0-2 vs. 3-6)

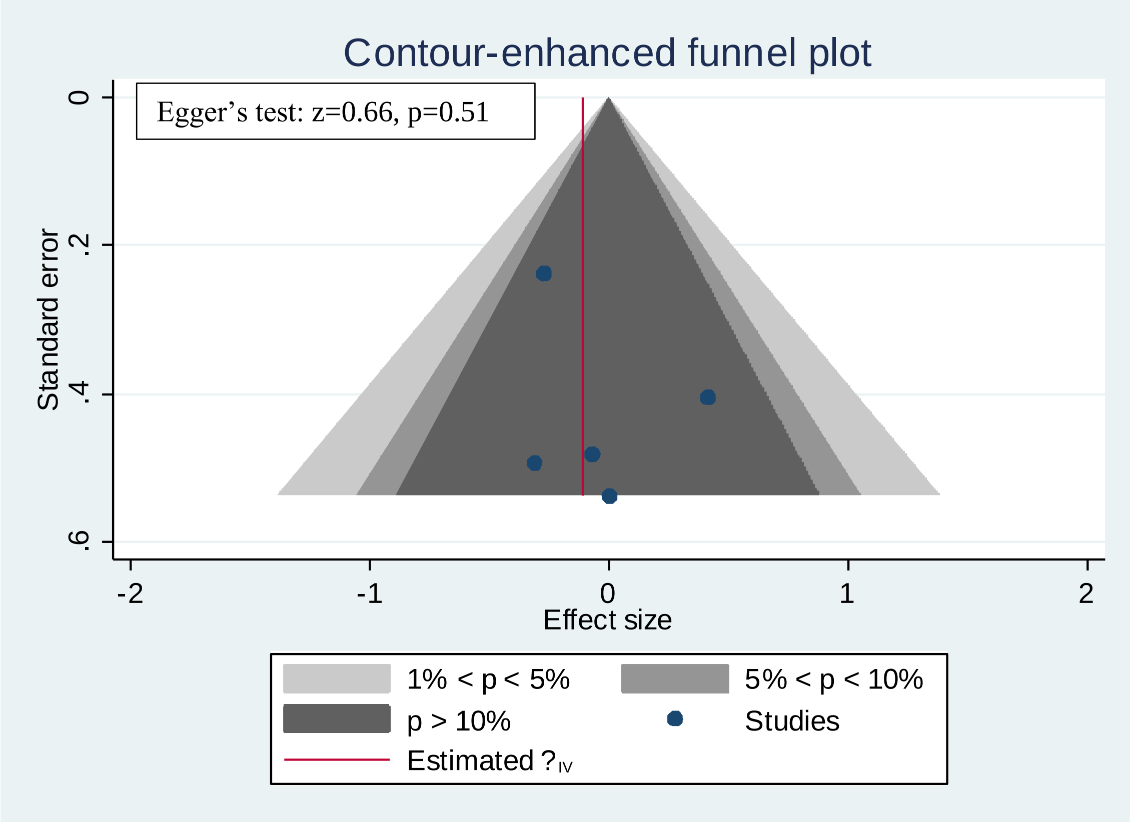

mRS denotes modified Rankin Scale
